# Role of the Hypoxic-Secretome in Seed and Soil Metastatic Preparation

**DOI:** 10.3390/cancers14235930

**Published:** 2022-11-30

**Authors:** Cynthia Clemente-González, Amancio Carnero

**Affiliations:** 1 Instituto de Biomedicina de Sevilla (IBIS), Consejo Superior de Investigaciones Científicas, Hospital Universitario Virgen del Rocío (HUVR), Universidad de Sevilla, 41013 Seville, Spain; 2 CIBERONC (Centro de Investigación Biomédica en Red Cáncer), Instituto de Salud Carlos III, 28029 Madrid, Spain

**Keywords:** cancer, metastasis, hypoxia, seed and soil, microenvironment, exosomes

## Abstract

**Simple Summary:**

During tumor growth the aberrant or absent vasculature causes hypoxia, an important decrease in the supply of oxygen to the cells that causes an adaptative response in the microenvironment of the tumor. Hypoxia activates the expression of genes that control several essential cellular processes. Among others, hypoxia induces the expansion of cancer stem cell (CSC) pools and promotes the transcription and secretion of factors that will adapt niches in different organs to receive, proliferate and promote the survival of malignant cells from the primary tumor, forming metastasis. CSCs at the secondary site also interact with stromal cells in the secondary organ to promote metastasis. Several cytokines released by cells within the secondary tumor microenvironment determine the tissue inflammatory infiltration and the cancer-associated phenotype of the immune component. Therefore, hypoxia initiates a cascade of physiological responses that not only affect the primary tumor, but also prepare secondary niches in distant organs for the apparition and development of metastasis.

**Abstract:**

During tumor growth, the delivery of oxygen to cells is impaired due to aberrant or absent vasculature. This causes an adaptative response that activates the expression of genes that control several essential processes, such as glycolysis, neovascularization, immune suppression, and the cancer stemness phenotype, leading to increased metastasis and resistance to therapy. Hypoxic tumor cells also respond to an altered hypoxic microenvironment by secreting vesicles, factors, cytokines and nucleic acids that modify not only the immediate microenvironment but also organs at distant sites, allowing or facilitating the attachment and growth of tumor cells and contributing to metastasis. Hypoxia induces the release of molecules of different biochemical natures, either secreted or inside extracellular vesicles, and both tumor cells and stromal cells are involved in this process. The mechanisms by which these signals that can modify the premetastatic niche are sent from the primary tumor site include changes in the extracellular matrix, recruitment and activation of different stromal cells and immune or nonimmune cells, metabolic reprogramming, and molecular signaling network rewiring. In this review, we will discuss how hypoxia might alter the premetastatic niche through different signaling molecules.

## 1. Introduction

Metastasis is the process by which tumor cells escape from the primary site and establish and reconstitute the tumor in a distant secondary organ. It is a process with multiple stages and is directed by multiple factors, such as organ-, tumor- and immune-related factors. To date, avoiding metastasis formation or eliminating it once it has appeared remains one of the main obstacles to achieve complete remission of cancer after treatment, and metastasis continues to be the leading cause of cancer-related death [1]. In order to accomplish metastatic colonization, the tumor cells that manage to enter the circulation must find a permissive environment to settle in that stimulates their survival and facilitates their adhesion. This concept, known as the seed and soil theory, was proposed by Stephen Paget as early as 1889 [2]. Therefore, aside from the adaptation of tumor cells to a new environment through tumor plasticity, it is reasonable to think that prior to releasing the tumoral cells into the circulation, the tumor remodels and adapts the secondary site to its needs by sending different factors, such as cytokines, chemokines, enzymes, and growth factors. This view of cancer metastasis as a bidirectional process in which both the disseminating cells and the secondary organ play a key role has been previously described and validated in several cancer types such as breast, colon and melanoma [3]. Recently, it has also been proposed that nasopharyngeal carcinomas may behave as pathological ecosystems, with self-seeding CTCs (Circulating Tumor Cells) releasing soluble factors and exosomes back to the primary tumor site [4]. In recent years, the hypothesis that the hypoxic environment that characterizes tumors may have a direct role in the preparation of the premetastatic niche has been gaining strength, with numerous results supporting it [5,6]. Hypoxia can carry out this function by inducing the release of various molecules, either secreted or inside extracellular vesicles, from both tumor cells themselves and stromal cells [7]. The mechanisms by which these signals that can modify the premetastatic niche are sent by the primary tumor include changes in the extracellular matrix, metabolic reprogramming, and recruitment and activation of different stromal cell types [8]. The present review focuses on the role of the hypoxic-secretome in the preparation of niches for metastasis growth in distant organs. Therefore, we establish a workflow for this review as: (1) hypoxic secretome, (2) hypoxia-related exosomes and (3) effect of hypoxia on cellular components of the premetastatic niche.

## 2. Hypoxic Secretome

**Figure 1:**
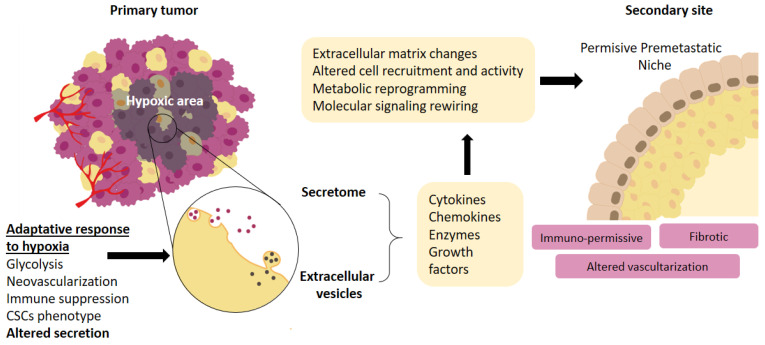
Schematic summary. As part of the adaptative response to hypoxic conditions, both tumoral and stromal cells alter the secretion of free molecules and extracellular vesicles, which upon arrival at the secondary site promote a series of changes that result in a permissive premetastatic niche.

The term “hypoxic secretome” refers to the set of molecules secreted into the extracellular space by a cell, tissue, organ, or organism in response to an environment with low O_2_ pressure [9]. Many of these molecules are secreted in exosomes or extracellular vesicles, but others may be liberated as free secreted factors, proteins, cytokines or RNAs (Figure 1).

Among the many molecules released into the environment by the primary tumor under these conditions, there are molecules related to growth and survival (HSP90a (Heat Shock Protein 90a), lncRNA-UCA1, osteopontin and periostin), formation of blood vessels (VEGF (Vascular Endothelial Growth Factor), SDF-1, TGF-β (Tumor Growth Factor β), ANG-1, FGF (Fibroblast Growth Factor), PDGF, and MMPs (Matrix Metalloproteinases)), immune evasion at both primary and secondary sites (CCL5, CXCL12 (Chemokine (C-X-C motif) Ligand 12), IL-10 (Interleukine 10), SDF-1, TGFβ1, VEGF, CCL2), migration (LOX (Lysyl Oxidase), MMPs), intravasation and extravasation (ANGPTL4, CCL2, IL-6, MMPs, PGF, VEGF) and metastatic colonization (LOX, tenascin C, PTHrP (Parathyroid Hormone-related Protein)) [10]. Additionally, many have several effects on the adaptation of the secondary site to make it a hospitable terrain for cells arriving from the primary site. In this review, we briefly describe the effect of the hypoxic secretome on the development of metastasis, with special emphasis on the role that some of these molecules play in establishing a favorable premetastatic microenvironment. See Figure 1 and Table 1.

### 2.1. Lysyl Oxidase

The lysyl oxidase (LOX) family of proteins is made up of a total of five enzymes (LOX and lysyl oxidase-like 1-4) whose expression can be induced by HIF in response to hypoxic conditions. Under physiological conditions, the function of these proteins is to carry out the oxidative deamination of lysine residues in different proteins [11].

According to a study carried out by Erler et al. in 2006, LOX appears to be essential in hypoxia-induced metastasis. Different microarray studies have shown an association between tumor hypoxia levels and LOX expression in tumors such as breast cancer and head and neck cancer. Furthermore, patients with elevated LOX levels show both poor overall survival and poor metastasis-free survival [12,13]. Increased hypoxia-induced LOX secretion is correlated with increased bone metastasis in ER-breast cancer [14]. In epithelial ovarian carcinoma, it is negatively correlated with progression-free survival [15], tumor grade, tumor diameter, and lymph node metastasis [16]. In gastric cancer, LOX levels are correlated with the number of lymph node metastases, greater infiltration depth, and advanced tumor-node-metastasis stages [17]. Elevated LOX expression in hepatocellular carcinoma is correlated with a higher relapse rate and worse survival rate [18]. In lung adenocarcinoma, LOX is part of the hypoxia-related risk signature [19], a correlation that is also observed in non-small cell lung carcinoma [20]. On the other hand, in breast cancer, the most frequent metastatic sites are the bone and the lung, and LOX has been proposed to play an essential role in the development of the premetastatic niche in those specific organs [21].

Therefore, it seems clear that there is a close relationship between LOX expression and secretion under hypoxia and the development of metastases. Recently, several studies have revealed some of the mechanisms through which LOX might exert this function. Some researchers hypothesize that it is due to its participation in migration and invasion via the LOX–FAK–AKT (Protein Kinase B) axis and in EMT (Epithelial to Mesenchymal Transition)via the E-cadherin pathway [22], while others suggest that it is through the establishment of a favorable premetastatic niche. The mechanism would be as follows: under hypoxia, highly metastatic cell lines such as MDA-MB-231 increase the release of nucleotides, specifically ATP, into the tumor interstitium. This ATP is then detected via P2Y2R, a G-protein coupled receptor, resulting in a higher transcription of HIF1α. Once HIF1α expression has been increased, it results in a further increased expression of LOX [23]. In recent years, it has been suggested that LOX may also be regulated by HIF2α, even more strongly than by HIF1α [24]. HIF then stimulates the expression of LOX, which is secreted and transported to a secondary site via the bloodstream. Once there, the enzyme proceeds to induce oxidative deamination of the lysine residues in collagen, allowing collagen crosslinking and the consequent formation of collagen IV fibers [25]. This increases the rigidity of the extracellular matrix and promotes the adhesion of cells that reach the secondary site [11,26]. In High Grade Serous Ovarian Cancer, there is a relationship between extracellular matrix remodeling induced by hypoxia-mediated LOX secretion and peritoneal metastasis [27]. Collagen IV crosslinking favors the recruitment of CD11b+ bone marrow-derived cells (BMDCs) [25,26,28]. Once recruited, BMDCs produce MMP2, which breaks down collagen. On the one hand, this rupture favors remodeling of the premetastatic niche, and on the other hand, it stimulates the arrival of even more BMDCs and tumor cells to the site. BMDCs also secrete angiogenic factors that promote vascularization of the BMDCs and facilitate the arrival of circulating tumor cells [25]. Moreover, when the matrix is stiffer due to collagen crosslinking, there is increased phosphorylation of the FAK/SRC (Steroid receptor coactivator) pathway, leading to the development of a more proliferative and invasive phenotype [29]. Once activated, the FAK/SRC pathway promotes the production of fibronectin. Fibronectin production recruits myeloid-derived suppressor cells, which contribute to the formation of an immune-suppressed premetastatic niche [30]. Finally, LOX promotes osteolytic activity in the bone, an essential event in the formation of premetastatic lesions in this organ [31]. These effects exerted by LOX that arrives via the bloodstream from the primary tumor come as no surprise, since LOX secreted by CAFs (Cancer Associated Fibroblasts) in the hepatic premetastatic niche in gastric cancer contributes to the formation of the niche and is correlated with a worse prognosis [32].

Physiological inhibition of LOX levels by miRNA reduces metastasis and improves prognosis. miRNA29a interacts with the 3’ UTR (Untranslated Region) of LOX, LOXL2 and VEGFA, reducing protein levels. A lower level of miRNA29a is associated with a worse prognosis [33]. Blocking LOX activity with synthetic inhibitors shows a similar effect. Inhibition of the HIF–LOX axis reduces metastasis in orthotopic models of breast cancer [20]. LOX inhibition also appears to block metastasis in pancreatic cancer [34]. The use of LOX inhibitors, such as Pdcd4 and β-aminopropionitrile, decreases the invasive capacity of the breast tumor cell lines T47D and MCF7 [35] and reverses LOX-induced EMT, invasion and metastasis in cervical cancer [36]. However, all these studies focused on demonstrating that LOX inhibition reduces metastatic capacity by preventing it from promoting epithelial–mesenchymal transition. Therefore, in the future, it is necessary to delve deeper into the effect that LOX inhibition has on the development of the premetastatic niche, since there is ample evidence of the role of LOX in this stage of metastasis.

Other proteins in this family, such as LOXL2, also seem to favor metastasis, although to date, studies have focused on pathways other than the formation of the premetastatic niche. For example, LOXL2 seems to promote lung metastasis in breast cancer, possibly by increasing epithelial–mesenchymal transition by stimulating the expression of Snail1 [37]. LOXL2, secreted in vesicles in response to hypoxia, not only acts in the premetastatic niche but can also reach cells of the primary tumor that are not under hypoxic conditions, which helps them carry out epithelial–mesenchymal transition and initiate invasion from nearby tissues. Moreover, the amount of LOXL2 present in extracellular vesicles is higher under hypoxic conditions than under normoxic conditions in head and neck cancer [30]. The secretion of LOXL2 and LOXL4 by breast cancer tumor cells results in increased crosslinking of collagen in the lung, one of the main sites of metastasis for this tumor type [28].

### 2.2. VEGF

VEGF is a growth factor that induces endothelial cell growth, angiogenesis, and vasculogenesis. VEGF can be induced by hypoxia, as it presents an HIF binding element in its promoter. The activity of VEGF in the neovascularization and permeabilization of blood vessels are its best known roles in metastasis, although it also participates in the establishment of the premetastatic niche [26].

Increased levels of VEGF expression in prostate cancer are associated with the clinical stage, Gleason score, tumor stage, progression, metastasis, and survival. Elevated plasma VEGF levels are correlated with bone metastasis and poor prognosis in many tumor types [38]. In addition, clinical data indicate that VEGFA and tumor-derived VEGFD induce prometastatic lymphangiogenesis and are associated with increased lymph node metastasis [39].

VEGF can exert its effect in preparing the premetastatic niche through different pathways. First, VEGF is capable of activating osteolysis in bone through the BMP (Bone Morphogenetic Protein) pathway. In addition, VEGF is capable of carrying out both autocrine and paracrine signaling in osteoblasts, resulting in the stimulation of bone resorption [38,40,41,42,43]. Osteoclasts are the main cells responsible for tumor-induced bone destruction. The resorption of the bone matrix has two different metastasis-promoting effects: it generates a physical space that can be occupied by tumor and stromal cells, and allows the release of growth factors that are trapped within it and that help generate a suitable environment for tumor growth [44,45]. In prostate cancer, activation of osteoblastogenesis is mediated by the VEGF/VEGFR (Vascular Endothelial Growth Factor Receptor) axis through BMP [41]. Another of its actions occurs through stimulation of the production of the proinflammatory cytokines S100A8 and S100A9 at secondary sites, such as the lung, by myeloid and endothelial cells that are already located there before metastasis [46]. In turn, these cytokines remodel the niche through different effects, such as the recruitment of CD11b+ myeloid cells [47]. VEGF also recruits VEGFR1+ BMDCs to fibronectin-rich sites (such as lung and bone). These BMDCs are an essential component in the formation of the premetastatic niche [43] and can stimulate the proliferation and metastasis of esophageal cancer cells in the niche [48]. Once there, BMDCs contribute to extracellular matrix remodeling through the secretion of MMP-9 [43,49]. There are eight pathways through which recruited BMDCs are estimated to contribute to lung bone metastasis: the ‘T-cell receptor signaling pathway’, ‘osteoclast differentiation’, the ‘MAPK signaling pathway’, the ‘VEGF signaling pathway’, ‘leukocyte transendothelial migration’, ‘signaling pathways regulating the pluripotency of stem cells’, the ‘oxytocin signaling pathway’ and ‘cell adhesion molecules’ (CAMs) [50]. On the other hand, VEGF seems to contribute to bone recognition by tumor cells and to the induction of their nesting [51]. It does so by mediating the expression of adhesion molecules, such as fibronectin and bone sialoprotein, in the extracellular matrix, which can be used by tumor cells to adhere to bone [52]. VEGF also results in increased CXCL5 expression in the lungs (alveolar epithelial cells), which is recognized by CXCR2 (C-X-C Chemokine Receptor) expressed by esophageal cancer tumor cells [48]. Furthermore, VEGF secreted by tumors in mouse mammary glands alters the premetastatic niche and triggers an inflammatory response, inducing the expression of prostaglandin E2 in mouse pulmonary microvascular endothelial cells (MPVECs). This increases the adhesion of breast cancer cells and promotes their organotropism to the lung [53], and increases the activation of invadopodia formation by circulating tumor cells via p 38, facilitating adhesion to the premetastatic site [54]. Finally, VEGF plays a key role in the structure of the secondary site vasculature. Neovascularization of the new environment is important not only to facilitate the arrival of circulating tumor cells but also because when circulating tumor cells reach the secondary site, they initially form a micrometastasis that becomes a macrometastasis, requiring neovascularization that allows them to obtain nutrients [55]. VEGF secreted by the primary tumor also increases the permeability of blood vessels in the premetastatic area by inducing Ser490 phosphorylation of occludin, mediating its degradation via the ubiquitin—proteasome pathway and therefore weakening the tight junctions in the blood vessels of the premetastatic niche in the lung in breast cancer [56].

### 2.3. TGF-β

TGF-β is part of a family of proteins that includes the three protein isoforms TGFβ-1, TGFβ-2 and TGFβ-3 and other functionally related proteins. These proteins play a key role in immune cells and endothelial and epithelial tissues by participating in processes such as survival, proliferation, differentiation, and migration [57]. TGF-β is regulated through hypoxia [58]. Hypoxia induces tumor cell secretion of TGF-β, and contributes to angiogenesis in several tumor types [59].

Blood TGF-β levels appear to be correlated with bone metastasis in prostate cancer patients [60]. Different mechanisms that lead to an increase in TGFβ pathway signaling have been identified as promoters of the formation of the premetastatic niche [57]. One of the more clearly characterized pathways seems to be that of the communication of ovarian cancer tumor cells with the premetastatic niche in the peritoneum. The delivery of TGF-β from the primary to the secondary site induces mesothelial-to-mesenchymal transition in the mesothelial cells of the peritoneum, which seems essential for the development of an adequate premetastatic niche [61]. Similarly, activation of the TGF-β signaling pathway in peritoneal mesothelial cells allows metastasis of gastric cancer to the peritoneum [62]. Osteosarcoma tumor cells send TGFβ packaged in EVs (Extracellular Vesicles) to lung fibroblasts, inducing their differentiation into cancer-associated fibroblasts, which contribute to generation of the premetastatic niche [63]. Activation of the TGFβ-SMAD2/3 pathway in Kupffer cells contributes to gastric cancer metastasis to the liver by causing Kupffer cells to promote activation of the CSC properties of incoming gastric cancer cells [64]. On the other hand, TGF-β induces the expression of CCL9 (Chemokine (C-C motif) Ligand 9) by CD11b+ myeloid cells. CCL9 fundamentally acts as a cell survival factor that allows the survival of tumor cells during the metastatic process. Furthermore, signaling of CCL9 through CCR1+ (C-C Motif Chemokine Receptor 1) results in the recruitment of myeloid progenitor cells that facilitate tumor cell invasion [65].

In addition, TGF-β promotes an immunosuppressive niche by inhibiting NK (Natural Killer) cells, γδ T lymphocytes, and CD8+ lymphocytes while promoting the polarization of neutrophils and macrophages to N2 and M2, respectively. TGF-β also stimulates CCR1+ myeloid cells, which secrete MMPs that remodel the premetastatic niche [66]. The effect of TGFβ on immune evasion during liver metastasis has been recently demonstrated by Taurellio et al. In their murine model, liver metastases exhibited high levels of TGFβ in the stroma along with a low rate of T-lymphocyte infiltration [67]. Blocking TGFβ signaling with inhibitors increased T-cell activity in clearing tumor cells and resulted in a reduction in the appearance of metastases in the early stages [67].

On the other hand, TGFβ secreted by Kupffer cells activates HSCs, causing the production and deposition of collagen-1 and fibronectin. This fibrous environment produces a greater recruitment of bone marrow-derived macrophages and granulocytes [68] while acting as a physical barrier that makes it difficult for the immune system to eliminate tumor cells [57]. In salivary adenoid cystic carcinoma metastases, vesicles secreted by the primary tumor containing α2β1 integrins are taken up by lung fibroblasts, causing them to secrete TGFβ. TGFβ increases the remodeling capacity of the microenvironment at the secondary site, in part through the production of periostin. Periostin is a protein that binds collagen and fibronectin and activates the AKT and STAT3 signaling pathways, thereby recruiting myeloid suppressor cells and creating an immunosuppressive environment in the secondary organ [69]. Finally, TGFβ1 produced by platelets in prostate cancer seems to affect the osteoclastogenesis/osteoblastogenesis balance, resulting in bone lesions that favor the development of a premetastatic microenvironment [70].Although many of these studies do not directly describe the role of TGFβ secreted by the primary tumor, they do seem to suggest that if TGFβ from other sources can have such a high impact on premetastatic niche formation, it could be expected that TGFβ secreted in response to hypoxia could act similarly, making it an interesting avenue for future research. 

### 2.4. CAIX

The carbonic anhydrase (CA) family of metalloenzymes includes numerous isoforms and is responsible for catalyzing the conversion of carbon dioxide to bicarbonate anion [71]. Under hypoxic conditions, HIF-1 translocates to the nucleus and activates the expression of carbonic anhydrase IX (CAIX) [72]. In cancer, the increase in CAIX levels in response to hypoxia is a mechanism of the tumor response to stress caused by low oxygen pressure. CAIX functions as a mediator of tumor growth and metastasis [73].

Multiple studies have shown a correlation between CAIX and poor prognosis or increased metastasis [74,75]. In thyroid cancer, patients with higher CAIX expression have a higher frequency of lymph node metastases [76]. The use of numerous combinations of CAIX inhibitors reduces the metastatic capacity of tumor cells in various models [77]. For example, CAIX promotes cell motility in cervical cancer through the ERK/PFKFB4 pathway [78], its inhibition in triple-negative breast cancer reduces lung metastasis [75], and suppression of the HIF1α/CAIX axis in epithelial ovary cancer reduces malignancy and invasion [79].

CA contributes to virtually all stages of tumor development and progression [80]. Regarding the ways in which CAIX participates in the development of metastases, CAIX levels are correlated with the expression of various integrins and MMP14, which are necessary for pseudopodia/invadopodia formation and degradation of the extracellular matrix at the beginning of the metastatic process [81]. CAIX overexpression increases MMP9 expression and FAK and steroid receptor coactivator (Src) phosphorylation, promoting tumor cell motility in oral squamous cell carcinoma [82]. However, currently, only one mechanism has been described through which CAIX seems to influence the establishment of the premetastatic niche. HIF1 promotes G-CSF production in breast cancer cells by activating the carbonic anhydrase axis CAIX-NFkB-G-CSF. G-CSF mobilizes granulocytic CD11b+Ly6G+Ly6C+ myeloid-derived suppressor cells (MDSCs) to the lung [83], where they produce Bv8 protein to induce premetastatic niche formation [84]. CD11b+Ly6G+Ly6C+ cells can also suppress IFNγ (Interferon γ) production and NK cell cytotoxicity in the niche [85]. Targeting G-CSF appears to reduce metastasis by partially preventing premetastatic niche formation [83]. G-CSF can be shipped within exosomes and is involved in increased angiogenesis and vascular permeability [86]. In addition, it is believed that G-CSF may play a role in the generation of metastatic lesions by cancer stem cells [87,88].

### 2.5. S100A8 and S100A9

S100A8 and S100A9 are two proinflammatory cytokines through which many of the molecules secreted by the primary tumor in response to hypoxia exert their function. For example, TNFα (Tumor Necrosis Factor α) and TGF-β, together with VEGFA, induce the expression of S100A8 and S100A9 in the lung, promoting development of the premetastatic niche [46].

Moreover, S100A8 and S100A9 induce the transcription of serum amyloid A (SAA) 3, which acts as a chemoattractant for Mac1+ myeloid cells in premetastatic sites through the activation of NKκB via TLR4 (Toll Like Receptor 4). However, the specific molecular mechanism remains unknown. The accumulation of Mac1+ at the premetastatic site generates an inflammatory-like state which accelerates the arrival of tumor cells. Furthermore, SAA3 stablishes a positive feedback loop by which it enhances its own secretion via TLR4 [46]. SAA3 also maintains a state of chronic inflammation at the secondary site [89] and plays an important role in metastatic niche formation [90]. SAA3 protein produced in response to S100A8 in the lungs also attracts and recruits circulating tumor cells to the niche [46] and stimulates the production of inflammatory cytokines, such as TNFα [89], which promote tumor growth [91]. S100A8 and S100A9 also recruit CD11b+ myeloid cells to the niche [54], where they deposit versican in the extracellular matrix [92] and secrete large amounts of MMP9 [93]. 

### 2.6. IL-6

Although IL-6 is often not considered in the effect of the hypoxic secretome on the formation of the premetastatic niche, there are multiple lines of evidence indicating that IL-6 could be a molecule of interest in future research in this area.

A recent study showed that the IL6/STAT3 pathway orchestrates the formation of an immunosuppressive premetastatic niche in the lungs. IL-6 deregulation in tumor cells reprograms the STAT3 pathway in metastatic cells and promotes the recruitment of myeloid suppressor cells and macrophage polarization toward a phenotype that allows tumor cells to escape immune system surveillance [94]. Furthermore, it was previously shown that breast cancer tumor cells secrete IL-6, which causes STAT3 Y705 phosphorylation in the lymphatic endothelial cells of lymphatic vessels. This phosphorylation activates HIF1α and VEGF and the expression of CCL5 in LECs (Lymphatic Endothelial Cells), directing the spread of the tumor to other tissues [95]. On the other hand, IL-6 is one of the main cytokines involved in the formation of the bone premetastatic niche through bone remodeling. Binding of IL-6 to its receptor activates the JAK/STAT pathway and promotes osteoblast differentiation towards a mature phenotype [96]. IL-6 secreted by both tumor cells and stromal cells is involved in the induction of osteoblastogenesis and bone resorption, which are key processes in the formation of the premetastatic niche in bone [97,98,99,100]. PC3 conditioned medium, rich in IL-6 and IL-8, induces the osteoclastic differentiation of CD11b+ blood cells and bone resorption [101]. Suppression of IL-6 expression in combination with clodronate liposome treatment in PC3 cells reduces osteoclast formation and reduces lymph node metastasis in a mouse model of metastasis [102]. Furthermore, the induction of IL-6 by macrophages under hypoxic conditions enhances the metastatic and invasive capacity of cancer cells [103]. Finally, the secretion of IL-6 by endothelial cells in the tumor microenvironment is also capable of activating MMP9 and causing tumor remodeling [104].

### 2.7. Other Molecules

The roles of other molecules secreted by the primary tumor in response to hypoxia in the formation of the premetastatic niche are not well known but would be interesting to consider (Table 1).

PTHrP secretion by breast cancer cells can be induced by HI2α [105], and this secretion is related to an increase in osteolytic lesions and bone metastases and to increased bone marrow colonization [106].

CXCL12 can be induced by hypoxia and is known to promote breast cancer dissemination via CXCR4 signaling [107]. Among the factors secreted by primary tumor cells in response to hypoxia, CXCL12 could be an interesting factor because other functions are known to be exerted in the premetastatic niche when CXCL12 is secreted locally. For example, when CXCL12 is secreted by endothelial cells, it attracts CXCR4-expressing cells into the CXCL12 gradient, facilitating intravasation [108]. On the other hand, the induction of CXCL12 expression in the premetastatic niche by factors secreted from the primary tumor is also involved in the homing of tumor cells to the secondary site. As an example, in prostate cancer, the transfer of pyruvate kinase 2 from the primary tumor to bone marrow stromal cells results in the increased expression of CXCL12 in the bone marrow in an HIF1α-dependent manner, favoring the establishment of metastatic cells in bone [109].

Matrix metalloproteinases constitute another interesting group of molecules. MMP9 is produced in response to VEGF and PIGF (Placenta Growth Factor) released by the primary tumor, and is responsible for breaking down the extracellular matrix to weaken the physical barrier that it constitutes, simultaneously releasing growth factors and soluble molecules that are trapped within it [49]. In addition, MMP13 is induced by HIF1α and is capable of breaking down pro-MMP9 to produce active MMP9 [5]. On the other hand, several of the molecules that arrive at the secondary site result in the recruitment of CD11b+ BMDCs. MMP9 secreted by VEGFR+ and VLA4+ (Very Late Antigen 4) BMDCs contributes to extracellular matrix remodeling during premetastatic niche formation [110]. Mac1+ Cd11b+ cells recruited to the niche by LOX also produce MMP2, which lyses collagen and releases collagen IV chemoattractant, thereby recruiting more CD11b+ cKit+ BMDCs and metastatic cells [7].

Other molecules, such as osteopontin, periostin, tenascin and CKB (Creatine Kinase Brain-type), may also play an important role in the development of the premetastatic niche, despite the little information that is available to date [10]. Periostin in metastatic niches serves to concentrate and present Wnt ligands, thereby inducing and maintaining the stem-like properties of founder cells that reach a metastasis site [7]. Tenascin C is secreted by hypoxic breast tumor cells and promotes lung colonization by altering the Notch and WNT signaling pathways [111]. Tumor cells secrete brain-type creatine kinase (CKB) in response to hypoxia as a mechanism of adaptation to metabolic stress under these conditions. CKB generates phosphocreatine, which can subsequently be used by tumor cells to obtain ATP [112].

In the upcoming years, new secreted molecules could be taken into consideration for their effect on premetastatic niche formation. GRP78, or 78-kDA glucose-regulated protein, is a protein belonging to a group of heat shock proteins related to the response to the unfolded protein response. Its expression is significantly increased through HIF1α (Hypoxia-Inducible Factor α) under hypoxic conditions [113]. This molecule has already been shown to promote an immunosuppressive tumor microenvironment after being secreted by tumor cells by regulating the production of cytokines by dendritic cells and macrophages [114]. Recently, a study by Chen et al. in 2020 showed that GRP78 participates in the development of immunotolerance in the premetastatic niche. This effect is mediated by the modulation of the activity of macrophages and dendritic cells, the suppression of liver-resident NK cells, and the induction of TGF-β expression [115]. Additionally, in 2020, miRNA92a (regulated by hypoxia through HIF1α) [116] was reported to be released by BMDCs recruited to the premetastatic niche in response to hypoxia. Once secreted, miRNA92a acts by inhibiting its target SMAD7, leading to increased TGFβ secretion by hepatic stellate cells. Elevated levels of this miRNA have been found in the serum of lung cancer patients. Therefore, it is possible that miRNA92a also plays a role in premetastatic niche adaptation, although more research is needed [117].

## 3. Exosomes

Communication between cells in the tumor microenvironment and the premetastatic niche can occur not only through the secretion of soluble molecules but also through the release of molecules inside extracellular vesicles, such as exosomes [118]. Exosomes are extracellular vesicles generated by the invagination and budding of the endosomal membrane and are later released during the fusion of multivesicular endosomes [119].

Communication via tumor-derived vesicles (TDVs) is well known to play a key role in metastasis development in several tumor types, including nasopharyngeal [120,121], oral [122], bladder [123], lung [124], prostate [125], breast [126], pancreatic [127], and ovarian [128] cancer. Furthermore, the secretion of tumor-derived exosomes into the bloodstream has previously been considered a promoter of the formation of the premetastatic niche [129]. For example, exosomes derived from melanoma cells reprogram progenitor cells from the bone marrow and promote vascular leakiness at the premetastatic niche [130].

In recent years, hypoxia has been demonstrated to affect both the number of extracellular vesicles produced by tumoral and stromal cells and the molecular composition of their cargos [131].

The content of TDEs (Tumor Derived Exosomes) can extensively change the landscape of the premetastatic niche. HIF1α is one the most relevant cargos found in these vesicles and can be sent from one cell to another inside vesicles without its DNA-binding and transcriptional activity being compromised. Delivery of HIF1α results in an alteration of EMT-associated proteins in the receptor cell, such as E- and N-cadherin [120]. Other important proteins for premetastatic niche progression that can be delivered via TDVs are matrix metalloproteinases: the presence of MMP-13, MMP14 and C4.4A within TDVs increased under hypoxia. Moreover, exosomal MMP-13 can increase the expression levels of vimentin while reducing E-cadherin expression in recipient cells [121]. In addition to matrix remodeling enzymes, several soluble factors have also been found inside TDEs, including TGF-β, TNF-α, IL-6, and IL-10, all of which play a key role in regulating cell migration [124,125].

Proteins do not constitute the sole cargo of TDEs: a plethora of noncoding RNAs can also be packed in these vesicles. HIF1α activation results in an enrichment of several miRNAs as exosome cargos, such as miR-21-3p, miR125b-5p and miR181d-5p in ovarian cancer [132] and miR-135b and miR-21 in melanoma [133] and PANC cells [134]. Once it reaches its target cell, miR-21 triggers prometastatic phenotype polarization [122]. The delivery of lncRNA-UCA1 by TDEs from bladder cancer cells also affects EMT [123]. In general, the levels of miRNA loaded into exosomes are significantly higher under hypoxic conditions than under normoxic conditions [122].

**Figure 2:**
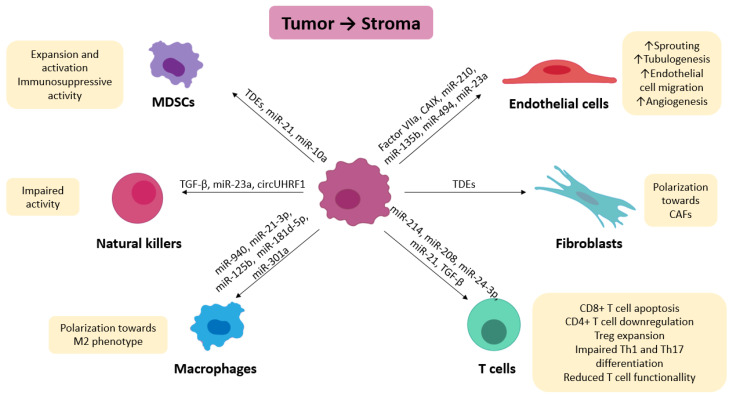
Exosomal communication of tumoral cells with stromal cells.

In the literature, hypoxia has been reported to induce the production of exosomes in different cancer cell lines, including breast cancer cell lines and oral squamous cell carcinoma cell lines [122,135]. Furthermore, several studies support the hypothesis that hypoxia increases the production of exosomes by tumoral cells compared to normoxic conditions [131]. Among the various ways in which HIF1α affects the production and release of exosomes, we found an upregulation of Rab7 and Rab27a via STAT3 [136], an increase in the expression of Rab22a and small GTPase Rab22a [126], an accumulation of Rab5 [137] and a rearrangement of cytoskeleton fibers through ROCK [138]. In addition, the presence of a hypoxic environment results in an increase in calpain expression, which has been suggested to cause the shedding of microvesicles [139]. Another effect of hypoxia is a reduction in the production of ceramide, which in turn promotes the biogenesis of ILVs, from which exosomes are then derived [140]. See Figure 2.

### 3.1. Effect of Hypoxic Exosomes on Stromal and Immune Cells

Tumor cells are not the only cells affected by the receipt of TDEs. The activity of stromal cells can also be affected by the receipt of several cargos [141].

TDEs have several effects on the biology of endothelial cells. They have been shown to play a role in the potentiation of angiogenesis [142] in cancer types such as glioblastoma (GBM), where hypoxic microvesicles containing tissue factor and factor VIIa promote endothelial cells to acquire an angiogenic phenotype [143] while stimulating microvessel sprouting. Furthermore, the release of carbonic anhydrase within TDEs from hypoxic renal cell carcinoma cells promotes endothelial cell migration and tube formation [86]. On the other hand, miR-210 secreted from leukemia cell lines induces tubulogenesis [144]; miR-135b inhibits FIH-1 (Factor Inhibiting HIF-1) and stimulates endothelial tube formation [133]; miR-494 from lung cancer cells targets PTEN, activating the AKT/eNOS pathway and resulting in enhanced angiogenesis; and miR-23a promotes angiogenesis by targeting prolyl hydroxylase and thus avoiding the degradation of HIF-1α [145].

In the case of fibroblasts, TDEs can promote polarization toward a cancer-associated phenotype (CAFs) in different ways, including the induction of α-SMA expression [125]. MicroRNA and proteins delivered by TDEs are internalized and result in the activation of several pathways that ultimately lead to a CAF-like phenotype [146]. However, a lot of specific information is missing on how this happens; thus the importance of highlighting the potential relevance of these processes.

TDEs also show the ability to generate an immunosuppressive environment and facilitate immune escape.

For example, TDEs can regulate T-cell activity by inducing CD8+ T cell apoptosis [147]. TDEs from S-180 cells and Lewis lung carcinoma cells downregulate CD4+ T cells and promote Treg expansion via miR-214 [148]. Treg expansion can also be achieved through the delivery of miR-208 inside exosomes [149]. Likewise, TDEs can induce Treg expansion and impair Th1 and Th17 differentiation by targeting fibroblast growth factor 11 due to miR-24-3p released within the vesicles [150]. Tumor exosomes reduce T cell cytotoxicity and proliferation through a mechanism that involves HSP70 [151]. The transfer of miR-21 within these vesicles amplifies the inhibitory effect that MDSCs have on T cells through the PTEN/PD-L1 pathway [151]. In addition, T cells that receive TGF-β from TDEs under hypoxic conditions lose their function [152].

The arrival of exosomes from cancer cells to macrophages induces polarization toward the M2 phenotype through the release of miR-940, miR-21-3p, miR125b, and miR-181d-5p and the blockade of PTEN/PI3Kgamma via miR-301a-3p [153]. The same is true regarding the delivery of miR-103a and polarization toward the M2 phenotype through activation of the AKT/STAT3 pathway [154].

The activity of NK cells is impaired in the hypoxic tumor microenvironment via several mechanisms [155]. Exosomal TGF-β secreted from lung cancer cells under hypoxia regulates the antitumoral response by decreasing the expression levels of NKG2D in NK cells, thereby reducing the activation of this cell type [156]. This exosomal TGFβ can also impair the cytotoxic activity of NK cells [157]. Moreover, miR-23a targets CD107a and consequently blocks NK cell activity [157], and circUHRF1 carried within exosomes is able to inhibit miR-449c-5p and, as a consequence, upregulate TIM3, which plays an immunosuppressive role [158].

In myeloid-derived suppressor cells, TDEs are able to promote MDSC immunosuppressive activity via heat shock protein Hsp72 [159] or Toll-like receptor 2 [160], and they also induce MDSC expansion and activation through miR-10a and miR-21 [161].

### 3.2. Effect of Hypoxic Exosomes from Stromal Cells on Tumor Cells

Exosomes derived from stromal cells have an impact on the behavior of tumor cells, making this cellular communication bidirectional. Hypoxic exosomes derived from macrophages deliver miR-223, which targets the Mef2c/β-catenin pathway and, as a result, promotes cancer invasiveness [162]. The delivery of apolipoprotein E by these vesicles is also able to promote cancer cell migration [163]. CAF-derived exosomes contain miR-21, which binds to APAF1 (Apoptosis Protease-Activating Factor-1) inside tumor cells and, as a result, suppresses apoptosis [164]. However, evidence that hypoxia effectively modifies CAF-derived exosome function is still lacking. The same is true for MSC-derived exosomes, for which very little evidence is currently available.

### 3.3. Effect of Hypoxic Exosomes from Stromal Cells on Stromal Cells

The effect of stromal-derived vesicles is also noticed in other stromal cells. As an example, the impact of DC-derived exosomes on other stromal cells is still being studied, and at the moment there is not enough evidence. Exosomes released by macrophages that contain miR-223 induce the differentiation of other monocytes toward macrophages [165]. Once again, there are not enough studies on MDSC-derived exosomes under hypoxia and their effect on other stromal cells.

## 4. Effect of Hypoxia on Cellular Components of the Premetastatic Niche

### 4.1. Effect of Hypoxia on Cellular Senescence and SASP Induction

Senescence is a cellular state characterized by an increase in p16 INK4A levels that results in cell cycle arrest [166]. It can be triggered by different stressors [167], including hypoxia [168].

Cellular senescence results in the increased expression and release of multiple secreted factors, which collectively are known as the senescence-associated secretory phenotype [169]. The factors secreted by senescent cells vary depending on the tissue and cell type, although at least 55 genes have been identified as common among all senescent cells [170], including VEGF, PDGF, HGF, IL1a, IL6, IL8, IL10, IL13, IL15, MMP3, MMP9, CXCL1, CXCL2, CXCL5, CXCL11, CXCL12, CCL2, and CCL20 [166].

The SASP (Senescence Associated Secretory Phenotype) can be divided into insoluble components, secreted proteases, and soluble signaling factors (interleukins, chemokines, and growth factors). Soluble factors include IL-6 [171], IL-1 [172], CCL2, G-CSF (Granulocyte Colony Stimulating Factor) [171] and GM-CSF (Granulocyte and Monocyte Colony Stimulating Factor). Among the secreted proteases, we can find several MMPs [169]. Insoluble components are released inside exosome-like small extracellular vesicles [173,174,175,176,177].

The expression of many SASP factors is regulated by activation of the NF-Kb and C/EBPbeta pathways during senescence [178,179]. Some of them also have positive feedback loops that regulate their expression, such as IL-1α, which regulates its own synthesis and is also a positive regulator of both IL-6 and IL-8 [180]. SASP secretion is also regulated by different miRNAs, such as miR-146a/b, which negatively regulate IL-6 and IL-8 secretion [181].

As we described in the previous paragraphs, many of these molecules have been associated with the preparation of the premetastatic niche. Furthermore, the SASP has several paracrine effects on neighboring cells, including promotion of angiogenesis [171], EMT, invasion [182] and metastasis [183]. Therefore, it is expected that the induction of senescence, and by extension the SASP, results in an enhanced PMN (Pre Metastatic Niche) preparation.

The role of IL-6 released by senescent cells has been particularly well described. First, IL-6 seems to have a key function in the induction of proliferation [184], EMT [185,186,187] and migration, most likely via STAT3 activation [188]. Breast cancer cell lines have been demonstrated to migrate more easily if cocultured with senescent cells [171]. This ability seems to be mediated by IL-6 and IL-8, both of which promote MMP expression [188,189]. Disruption of the basal membrane by matrix metalloproteases also enhances invasion and metastasis [174]. Additionally, IL-6 disrupts cell adhesion, thereby promoting invasion [190]. IL-6 also promotes tumor angiogenesis [191].

On the other hand, we have serum amyloid A (SAA), which is an acute phase protein that the liver secretes after injury or infection, although in recent years, different studies have reported that it can also be produced by cancer cells and cancer-associated cells. In this case, the production of SAA promotes cancer progression and metastasis [192]. SAA transcription is induced by the JAK/STAT3 pathway upon the interaction of IL-6 with its receptor. Alternatively, the binding of TNFα, IL-18 or IL-1β to their corresponding receptors also results in increased SAA expression via the NFkB pathway. Furthermore, IL1β displaces a constitutive repressor of NFkB signaling, further increasing the overexpression of SAA [193]. Notably, both IL-6 and IL-1b are molecules produced by senescent cells as part of the SASP. In turn, SAA expression results in the production of several proinflammatory cytokines by receptor cells [193]. SAA boosts the invasiveness capacity of tumor cell lines. For example, in renal cell carcinoma cell lines, SAA promotes MMP9 expression, and in glioma cell lines it induces the secretion of not only MMP9 but also of IL-8 and ROS (Reactive Oxygen Species) [194,195]. Several studies have noted that SAA might execute its role in cancer progression through autophagy regulation, potentially through the PI3K/AKT and MAPK pathways [192].

Fibronectin (FN) overexpression is likewise associated with senescence triggered by external stressors, such as hypoxia [196]. Furthermore, the folding of this protein might be compromised due to several factors, which also include hypoxia [197]. FN seems to promote metastasis [198] and is a well-known marker of the mesenchymal phenotype, which points toward the possibility that HIF1α overexpression under hypoxia might result in re-expression of FN in tumor cells, which promotes EMT. However, it is still not clear whether FN expression is a result or a consequence of metastatic initiation [199,200]. Matrix stiffness can seriously affect the metastatic process [201], and the following hypothesis has been proposed: FN allows a softer ECM that facilitates tumor vascularization for tumor growth until hypoxia slowly erases FN and stiffens the ECM upon the crosslinking of collagen induced by LOX. This promotes vascular abnormalities and therefore enhances metastasis [202,203]. This fact might seem contradictory to the previous affirmation that ECM destruction facilitates metastasis. However, both processes are not mutually exclusive, nor dependent on each other. The destruction of the ECM (specifically on the bone) generates a physical space that can be more easily occupied by the arriving cells. Furthermore, the destruction of ECM also releases molecules that were trapped inside and that may act as chemoattractants or nutrients. Once the cells arrive in the premetastatic niche, if the remaining ECM is stiffer than usual, it will be easier for the circulating tumor cells to attach to it.

FN may also facilitate secondary site colonization: CTCs become more resistant to anoikis by forming clusters in a process mediated by plakoglobin [204,205,206]. Plakoglobin stabilizes FN mRNA, facilitating periFN assembly on primary tumor cells and therefore cluster formation [207]. Last, but not least, tumor cells from the primary site secrete chemoattractants and exosomes containing stimulatory factors that likely increase FN expression in recruited TAMs (Tumor Associated Macrophages) and CAFs, which results in ECM deposition and remodeling [43,208]. This results in VEGFR1+ BMDC recruitment and adhesion to FN through integrin α4β1 expressed on their surface [43]. Apparently, protumor inflammatory chemokines and cytokines are characteristics shared among the TME, metastatic niche and premetastatic niche, resulting in the accumulation of polymerized FN that promotes tumor cell growth [54].

Finally, many of the SASP components act as regulators of the immune system and, as a consequence, have a remarkable effect on cancer progression. While the SASP may promote clearance of senescent cells and improve immunosurveillance when acutely activated [209,210,211], the chronic presence of the SASP promotes invasion and metastasis [182] due to the establishment of an immunosuppressive microenvironment [212].

The role of the SASP in premetastatic niche preparation has been described for several cancer types. For example, senescent osteoblasts that secrete IL-6 cause increased bone resorption, which leads to a more suitable environment for breast cancer cells to metastasize [213]. This suitability is most likely achieved through the recruitment of myeloid-derived suppressor cells by IL-6 [214]. In pancreatic cancer, FGF seems to drive cancer dissemination [215,216], and in breast cancer, IL-6 and IL-8 released by senescent fibroblasts increase invasiveness in several cell lines [171]. In addition, MMP2 and MMP3 secretion promotes invasion [169]. IL-1 activates the endothelium, thus increasing the ability of cancer cells to attach to blood vessels [217]. Finally, SASP components might be of particular interest due to their potential role in cancer cell dissemination in ovarian cancer, specifically through the expression of IL-6, IL-8 and MMPs. IL-6 increases the number of ovarian CSCs [218], stimulates EMT [219], and acts as an immunosuppressive cytokine [220]. On the other hand, IL-8 facilitates EMT, and high levels of this cytokine are correlated with greater cancer dissemination [221].

### 4.2. Effect of Hypoxia on CSCs

CSCs constitute a small subpopulation of cancer cells that present a range of stem cell-like properties, such as self-renewal capacity, quiescence, and a slower cell cycle [222,223]. They are responsible for processes related to poor prognosis, such as therapy resistance, metastasis and tumor relapse [224].

CSCs actively remodel their niche by secreting a range of factors that recruit other cells and modify the extracellular matrix characteristics [225]. In 2020, Lopez de Andrés et al. compiled a summary of the principal CSC secretome components that regulate those processes [224]. Among the molecules that constitute the CSC secretome, we can find several interleukins (IL-6, IL-8, IL-1β), multiple MMPs and VEGF, all of which may be secreted either in a soluble form or inside tumor-derived vesicles [226,227,228]. Other molecules, such as HIF1 [120], TGF-β [229,230,231], CCL2 [232] and miRNAs [233,234], are released into the bloodstream encapsulated inside exosomes.

The CSC secretome is able to interact with and modify the premetastatic niche at distal sites [234]. As an example, VEGF enhances the permeability of blood vessels surrounding the PMN [235]. Moreover, VEGF is also able to increase MMP-9 expression in cells in the secondary organ, which helps remodel the ECM [49]. In addition, BMDCs can be recruited by VEGF signaling [43]. Once they arrive, the BMDCs respond to TGFβ signaling by releasing CCL9, which helps generate a protumoral microenvironment [65]. Both VEGF and TGFβ stimulate the production of several inflammatory chemoattractants in the secondary site [54]. On the other hand, CSCs are known to show high levels of CXCR4 on their surface, which helps them recognize CXCL12. As a consequence, CXCR4/CXCL12 signaling may guide the migration and colonization of the secondary organ [236,237,238].

Hypoxia activates the transcription of HIF-1α and HIF-2α in CSCs [239,240], which in turn promotes the transcription of IL-6 and IL-8 [241,242]. IL signaling is a key factor in the upregulation of pathways such as Wnt/β-catenin, NFkB and SMADs, which are all required for the expansion of CSCs [218,243,244]. Furthermore, signaling by TGF-β, which is known to be secreted under hypoxia, has been demonstrated to activate IL-8 production by CSCs, resulting in an enrichment of that cell subpopulation [245]. Moreover, the secretion of several molecules encapsulated in exosomes is enhanced under hypoxic conditions and has also been reported to promote PMN formation [85,246]. Therefore, hypoxia might also contribute to PMN development by enhancing the survival and colonization chances of CSCs that leave the primary tumor and arrive at the secondary site.

CSCs from the primary tumor can be found in the premetastatic niche in both dormant and active states. The transition from one state to the other is determined by the stimuli received by the CSCs in the premetastatic niche [237]. Dormancy has been described as a common feature of cancer cells derived from a hypoxic microenvironment, and IL-6 expression has been reported to be related to a dormant phenotype [247]. The expression of thrombospondin in the perivascular niche maintains CSCs in a dormant state, and the production of tenascin C and fibronectin by endothelial cells accelerates the growth of CSCs [248]. CSCs can induce the production of these molecules by stromal cells, and once they receive their signal, pathways such as the Wnt, Nanog and Oct4 pathways are activated, allowing the CSCs to leave the dormant state [249,250]. Another way in which CSCs can leave the dormant state is through the action of fibronectin and type I collagen, which stimulate the ERK/MAPk pathway and beta1-integrin signaling [251,252]. The production of these molecules (tenascin C, fibronectin, etc.) that allow CSCs to leave the dormant state has been related to hypoxia, suggesting that hypoxia may also promote metastasis development by allowing CSCs at the secondary site to leave the dormant state.

CSCs at the secondary site also interact with stromal cells in the secondary organ to promote metastasis. Several cytokines released by cells within the tumor microenvironment determine the polarization of tissue macrophages, polarizing them towards a cancer-associated phenotype [253,254]. Some of these signaling molecules come from the cancer cells themselves [255]. After being polarized, tumor-associated macrophages (TAMs) secrete IL-6 [256], which binds to its receptor on CSCs and activates the STAT3 pathway, promoting CSC expansion and activation of the expression of stem-related genes, such as Sox-2, Oct-3/4 and Nanog. This has been verified in breast cancer stem cells and lung cancer cells [257]. The arrival of IL-6 also enhances breast CSC migration, promotes tumor growth and metastasis [258], and induces angiogenesis by inducing the expression of proangiogenic molecules, such as VEGF, MMP2 and MMP9 [259,260]. As another example of this type of interaction, breast cancer cells that arrive in the bone secrete osteopontin, which causes the differentiation of bone resident fibroblasts into myofibroblasts, promoting cancer progression [260]. The secretion of osteopontin also polarizes fibroblasts toward the CAF phenotype, and in turn, the CAFs release periostin to support cancer growth in the metastatic niche [237,249].

This evidence suggests that CSCs may have a revealing role in the formation of the PMN, especially when stimulated by a hypoxic environment in the primary organ, although further research is clearly needed if we are to understand the underlying mechanisms of this complex process [224].

## 5. Conclusions

**Figure 3:**
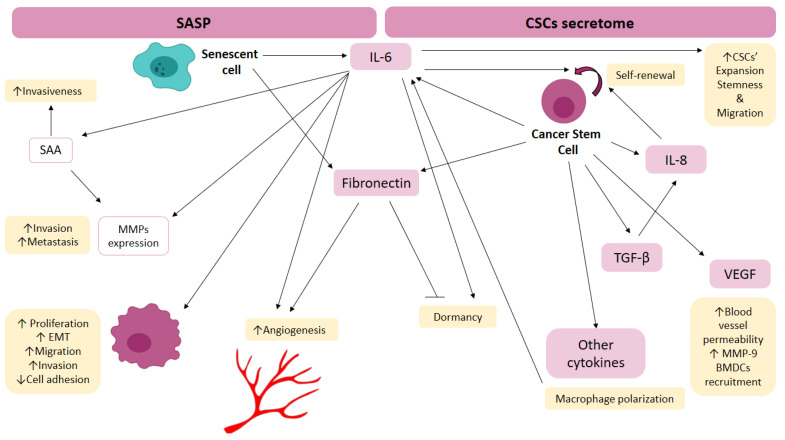
Effect of hypoxia on cellular components of the premetastatic niche.

During tumor growth, the delivery of oxygen to cells is hamperd by aberrant or absent vasculature that results in hypoxia. This causes an adaptative response that activates the expression of genes that control several essential processes. Hypoxia activates the transcription of HIF-1α and HIF-2α, which in turn induces CSC expansion by promoting the transcription of secreted proteins, citokines, non-coding RNAs and chemokines that will promote the adaptation of niches in different organs to receive, promote survival and adapt to proliferate malignant cells from the primary tumor, especially CSCs capable of regenerating the bulk of the tumor mass in the new emplacement, forming metastasis (Figure 3). CSCs at the secondary site also interact with stromal cells in the secondary organ to promote metastasis. Several cytokines released by cells within the secondary tumor microenvironment determine the tissue inflammatory infiltration and the cancer-associated phenotype of the immune component. Therefore, hypoxia initiates a cascade of physiological responses that not only affect the primary tumor, but also prepare secondary niches in distant organs for the apparition and development of metastasis.

## Figures and Tables

**Table 1:** Summary of the role of several hypoxia-induced molecules in the generation of the premetastatic niche.

Molecule	Effect on Premetastatic Niche Preparation		
	ECM (Extracellular Matrix) Changes	Cell Recruitment and Activity	Vascularization
LOX	Collagen crosslinking ↑Matrix stiffness ↑Fibronectin ↑Osteolysis	CD11b+ BMDCs and MDSCs recruitment ↑Proliferative and invasive phenotype	↑Angiogenesis
VEGF	↑Fibronectin ↑Osteolysis and bone reabsortion ↑Bone sialoprotein production MMP-9 secretion	CD11b+ BMDCs and VEGFR+ BMDCs recruitment CXCR2+ tumor cells recruitment Nesting induction ↑Adhesion ↑Invadipodia formation	↑Angiogenesis ↑Permeabilization
TGFb	Fibronectin, collagen and periostin production ↑Bone lesions	MDSCs recruitment Mesothelial to mesenchymal transition Immune suppression of NKs, d T cells and CD8+ T cells N2 and M2 polarization CCL9 expression in CD11b+ BMDCs	/
CAIX	↑Intregin expression ↑MMP-14 expression ↑MMP-9 secretion	↑Cell motility IFNg production suppression G-CSF production Inhibits NK citotoxicity	/
S100A8 and S100A9	MMP-9 secretion Versican deposit	CD11b+ BMDCs recruitment Invasive phenotype Increased invadipodia formation Chronic inflammation	/
SAA3		Mac1+ recruitment Circulating tumor cells recruitment ↑ TNFa production Chronic inflammation	/
IL-6	Osteolysis and bone reabsorption MMP-9 secretion	MDSCs recruitment CD11b+ osteoclastic differentiation Immunosuppresive niche	CCL5 expression in LECs
**Global** **effect**	Creates physical space Releases trapped molecules Increases cell adhesion and nesting Difficult access to eliminate tumor cells	Immune suppressed secondary site Increased tumor cell arrival	Favors circulating CSCs arrival Nutrient arrival to micrometastasis

## Abbreviations

AKT	Protein Kinase B/AKT
APAF1	Apoptosis Protease-Activating Factor-1
BMDCs	Bone Marrow-Derived Cells
BMP	Bone Morphogenetic Protein
CAFs	Cancer Associated Fibroblasts
CAIX	Carbonic Anhydrase IX
CAMs	Cell Adhesion Molecules
CCL9	Chemokine (C-C motif) Ligand 9
CCR1	C-C Motif Chemokine Receptor 1
CKB	Creatine Kinase Brain-type
CSCs	Cancer Stem Cells
CTCs	Circulating Tumor Cells
CXCL	Chemokine (C-X-C motif) Ligand 20
CXCR	C-X-C Chemokine Receptor
ECM	Extracellular Matrix
EMT	Epithelial to Mesenchymal Transition
EV	Extracellular Vesicles
FGF	Fibroblast Growth Factor
FIH-1	Factor Inhibiting HIF-1
FN	Fibronectin
GBM	Glioblastoma Multiforme
G-CSF	Granulocyte Colony Stimulating Factor
GM-CSF	Granulocyte and Monocyte Colony Stimulating Factor
GRP78	78-kDA glucose-regulated protein
HIF	Hypoxia-Inducible Factor
HSP	Heat Shock Protein
IFN	Interferon
IL	Interleukine
LECs	Lymphatic Endothelial Cells
LOX	Lysyl Oxidase
MDSCs	Myeloid-Derived Suppressor Cells
MMP	Matrix Metalloproteinase
MPVECs	Microvascular Endothelial Cells
NK	Natural Killer
PIGF	Placenta Growth Factor
PMN	Pre Metastatic Niche
PTHrP	Parathyroid Hormone-related Protein
ROS	Reactive Oxygen Species
SAA	Serum Amyloid A Protein
SASP	Senescence Associated Secretory Phenotype
Src	Steroid receptor coactivator
TAMs	Tumor Associated Macrophages
TDEs	Tumor Derived Exosomes
TDVs	Tumor Derived Vesicles
TGF-β	Tumor Growth Factor beta
TLR	Toll Like Receptor
TNFα	Tumor Necrosis Factor alpha
UTR	Untranslated Region
VEGF	Vascular Endothelial Growth Factor
VEGFR	Vascular Endothelial Growth Factor Receptor
VLA4	Very Late Antigen 4
